# Oral health and health-related quality of life in HIV patients

**DOI:** 10.1186/s12903-018-0605-4

**Published:** 2018-08-29

**Authors:** Vinicius da Costa Vieira, Liliane Lins, Viviane Almeida Sarmento, Eduardo Martins Netto, Carlos Brites

**Affiliations:** 1 School of Medicine, Federal University of Bahia, Praça XV de Novembro, Largo do Terreiro de Jesus s/n, Salvador, Bahia CEP 400260-10 Brazil; 2 School of Dentstry, Federal University of Bahia, Salvador, Bahia Brazil; 3 Research Laboratory of Infectious Diseases, Edgard Santos Federal University Hospital, Salvador, Bahia Brazil

**Keywords:** Health-related quality of life, Oral health, Depression, HIV

## Abstract

**Background:**

Oral health care may improve the health-related quality of life (HRQoL) of HIV/AIDS patients. We aimed to evaluate oral health and HRQoL of HIV/AIDS patients using antiretroviral therapy.

**Methods:**

A cross-sectional study included 120 HIV-infected patients, aged ≥18 years, from February, 2016 to September, 2017. The 36-Item Short Form Health Survey (SF-36) was used to evaluate the HRQoL. We assessed dental caries status using the Decayed, Missing and Filled Teeth (DMFT) index. Information about demographic, socioeconomic status, depression, and other comorbidities were collected. All patients with depression had a medical diagnosis. Comorbidities were defined as medical diagnoses of arterial hypertension, type-2 diabetes, tuberculosis, syphilis, cardiopathy, chronic renal failure, lymphoma, HCV infection, HBV infection and fatty liver disease. Independent t-tests were used to compare differences between mean levels of HRQoL, age, and DMFT and its components according to groups of sex, comorbidities and depression. Simple linear regression was used to analyze the relationship between the Mental Component Summary (MCS) and DMFT, and a multiple regression equation investigated depression, age, MCS, and comorbidities as predictors of DMFT.

**Results:**

The mean DMFT index was 12.4 ± 8.2. A linear regression equation estimated a significant (*p* = 0.022) decrease of 0.25 unit (%) in MCS for each unit increase in DMFT. Among depressed patients, a significant (*p* = 0.008) decrease of 0.67% in MCS for each unity increase in DMFT was estimated. Depressed patients showed worse oral health indicators (DFMT index; *p* ≤ 0.001; and mean Missing Teeth; *p* ≤ 0.052) and lower HRQoL domains than non-depressed patients. DMFT remained associated with depression (*P* < 0.005) after controlling for age, MCS, and comorbidities.

**Conclusions:**

We found association between poorer oral health (higher DMFT index) and lower Mental Health Component Summary in HIV-infected patients with depression. Patients with depression deserve especial attention to their HRQoL and oral care.

## Background

The proper use of antiretroviral therapy (ART) has extended the life expectancy of people living with HIV/AIDS [1]. In consequence, several health-related outcomes have been observed, that contributed to a higher frequency of chronic comorbidities [2–4], depression and depressive symptoms [5, 6] that lead to a poorer health-related quality of life (HRQoL) [3, 6–8] and increases the risk of low adherence to ART [9]. Assessing depression symptoms before initiating ART may be effective to improve adherence and characterize the health-related quality of life of these patients [10]. The assessment of the health-related quality of life became an integral part of HIV/AIDS patients’ follow-up [4].

In the past, detection of oral health lesions were often useful in the clinical diagnosis of HIV/AIDS infection, particularly among immunosuppressed patients [11]. There is no consensus about the association of poor oral health, particularly measured by DMFT index, with HIV infection. Some reports have shown greater risk for development of dental caries in HIV patients during antiretroviral drugs treatment [12, 13]. Another study reported a decrease in the incidence of dental caries following antiretroviral therapy [14]. A study among HIV-positive patients reported higher levels of immune activation markers HLA-DR and CD38 expressions in the peripheral blood when oral lesions were present [15], suggesting that oral health may significantly impact the immune response of HIV patients, including those under suppressive ART. However, HLA-DR and CD38 levels did not vary substantially according to the DMFT index.

Oral health care may improve the HRQoL of HIV/AIDS patients. A study in HIV patients has reported higher DMFT index associated with poorer Oral Health–Related Quality of Life [16]. However, this study did not investigate the association between DMFT index and the SF-36 domain scores. The SF-36 questionnaire is a widely used instrument to evaluate HRQoL, based on two different constructs, the Physical Component and the Mental Component [17]. This study aimed to evaluate the oral health and HRQoL of HIV/AIDS patients in use of ART.

## Methods

### Study design and participants

This is a cross-sectional study of HIV-infected patients, aged 18 years or more, consecutively recruited at the HIV Clinic of the University Hospital Professor Edgard Santos, Federal University of Bahia, Salvador, Bahia, Brazil, between February 2016 and September 2017. We excluded from the study patients unable to communicate or who had difficulty to understand SF-36 questionnaire.

### Assessment

Information about demographic, socioeconomic status, clinical history, HIV-1 RNA plasma viral load and CD4/CD8 cells count were collected from each patient during medical examination, using a structured questionnaire. The 36-Item Short Form Health Survey (SF-36) was used to evaluate the HRQoL^17^. We used the SF-36 as recommended by QualityMetric Incorporated [17] to generate eight domains - physical functioning (PF), role limitations due to physical problems (RP), bodily pain (BP), general health perceptions (GH), vitality (VT), social functioning (SF), role limitations due to emotional problems (RE) and mental health (MH). The raw score of these domains varies from 0 to 100, where 100 represents the best HRQoL. SF-36 scores were normalized, assuming a mean of 50 and a standard deviation of 10, taking the general population of the USA as standard. The normalized domains were aggregated into either Physical Component Summary (PCS) or Mental Component Summary (MCS) [17]. Our study was licensed by QualityMetric Health Outcomes™ under number QM025905.

We used the World Health Organization and the European Association of Dental Public Health criteria for oral health status evaluation [18, 19]. We measured clinical attachment loss, probing pocket depth and tooth mobility to evaluate periodontal disease. The number of Decayed, Missing and Filled Teeth were determined. Cariogenic diet was accessed using open *questions in the structured questionnaire*. The same researcher has evaluated all patients. The *intrarater* reliability (k = 0.67) was substantially satisfactory, as measured by the Kappa statistics [20]. Stimulated salivary flow measured less than 1 mL/min was considered as reduced [21].

### Statistical analysis

Health-related quality of life (HRQoL) [17] and the number of Decayed, Missing and Filled Teeth (DMFT index) [18] were considered as dependent variables. Independent t-tests were used to compare differences between mean levels of HRQoL, age, and DMFT and its components according to groups of sex, comorbidities and depression. Simple linear regression technique was used to analyze the relationship between MCS and DMFT, and a multiple regression equation investigated age, depression, MCS, and comorbidities as predictors of DMFT. All patients with depression had a medical diagnosis. Comorbidities were defined as medical diagnoses of arterial hypertension, type-2 diabetes, tuberculosis, syphilis, cardiopathy, chronic renal failure, lymphoma, HCV infection, HBV infection and fatty liver disease. Data were analyzed by using the Statistical Package for the Social Sciences 18 (SPSS).

### Ethical procedures

The study was approved by the Ethics Review Board of University Hospital Professor Edgard Santos, Federal University of Bahia under the Certificate of Presentation of Ethical Appreciation (CPEA 57172216.2.0000.0049) in accordance with the Declaration of Helsinki 2013 and the National Council Resolution 466/12 and. All participants were informed and signed a consent form approved by the Ethics Board.

## Results

The study enrolled 120 patients (64 males; 56 females). Age ranged from 20 to 72 years, and the mean (±SD) was 44.9 ± 11.7 years. Most were Mulatto (57.5%) or Black (24.2%) and were not in a stable relationship (73.3%). Thirty-six (30.0%) of the patients had elementary schooling level (four or less years); 70.8% were non-smokers; 65.0% consumed alcohol, and 28 (23.3%) had diagnosis of depression.

Arterial hypertension was present in 22 (18.3%) patients, type-2 diabetes in seven (5.8%), tuberculosis in eight (6.7%), syphilis in six (5.0%), cardiopathy in four (3.3%), chronic renal failure in two (3.5%) and lymphoma in one (0.8%). Of the patients with hepatic comorbidities, five (4.2%) had HCV, three (2.5%) HBV and three (2.5%) had fatty liver disease. Periodontitis and gingivitis were found in 52 (46.4%) and 47 (42.0%) of dentate patients, respectively. Twenty-four patients (20.0%) had reduced salivary flow; and the mean DMFT index was 12.4 ± 8.2 (1.1 ± 1.9 decayed teeth; 7.9 ± 8.7 missing teeth and 3.4 ± 4.0 filled teeth). Eighty patients (66.7%) had CD4 counts equal or greater than 500 cells/mm3 and 75 (62.5%) had undetectable viral load (Table 1).

**Table 1:** Demographic and clinical characteristics of 120 HIV-infected patients, Salvador, Bahia, 2017

Demographic and clinical characteristic	
Age, mean SD	44.9 ± 11.7
Sex N (%)	
Male	64 (53.3)
Female	56 (46.7)
Marital status N (%)	
Single	88 (73.3)
Married/stable relationship	32 (26.7)
Ethnicity N (%)	
Caucasian	22 (18.3)
Mulatto	69 (57.5)
Black	29 (24.2)
Educational status N (%)	
Elementary	36 (30.0)
High School	66 (55.0)
College	18 (15.0)
Alcohol consumption N (%)	
Yes	78 (65.0)
No	42 (35.0)
Smoking status N (%)	
Non Smoker	85 (70.8)
Smoker	35 (29.2)
Comorbidities N (%)	
Yes	62 (51.7)
No	58 (48.3)
Depression N (%)	
Yes	28 (23.3)
No	92 (76.7)
Daily dental brushing N (%)	
≥ 3 times	58 (48.3)
< 3 times	62 (51.7)
Dental floss use N (%)	
Yes	51 (42.5)
No	69 (67.5)
Cariogenic diet N (%)	
Yes	65 (54.2)
No	55 (45.8)
Edentulism N (%)	
Dentate	112 (93.3)
Edentulous	8 (6.7)
Periodontal disease	
Periodontitis	52 (46.4)
Gingivitis	47 (42.0)
No periodontal disease	13 (11.6)
DMFT, mean SD	12.4 ± 8.2
Decayed, mean SD	1.1 ± 1.9
Missing, mean SD	7.9 ± 8.7
Filled, mean SD	3.4 ± 4.0
CD4 cells/mm3, mean SD	656 ± 363
CD8 cells/mm3, mean SD	1008 ± 486
CD4/CD8 ratio	0.75 ± 0.44
Viral Load, mean SD^a^	35,245 ± 135,993
Viral Load, geometric mean SD^a^	309.3 */÷ 17.1

^a^Only patients with viral loads > zero

Patients with comorbidities were older (47.2 ± 11.0 vs. 42.4 ± 11.9; *P* = 0.026), and presented higher mean DMFT (14.0 ± 7.9 vs. 10.7 ± 8.2; P = 0.026). All SF-36 normalized mean scores were systematically lower in patients with comorbidities. Among patients with comorbidities, means of all SF-36 domains were significantly lower (*P* < 0.05), except for MH (Table 2). Women showed SF-36 scores systematically lower than men in all domains, and means of RP (0.006), BP (0.016), VT (0.044), MH (0.001) and MCS (0.013) were significantly lower. Compared to males, females presented higher mean DMFT (*P* < 0.001) and mean Missing Teeth indexes (*P* < 0.033) (Table 3).

**Table 2:** Mean and Standard Deviations of characteristics (Age, Oral Health Profile, and Health-related Quality of Life) of 120 patients according to comorbidities, Salvador, Bahia, Brazil, 2017

Characteristics	With Comorbidity (*N* = 62	Without Comorbidity (*N* = 58)	Mean Difference	P≤*
Age	47.2 ± 11.0	42.4 ± 11.9	4.8	0.026
DMFT Index	14.0 ± 7.9	10.7 ± 8.2	3.3	0.026
Decayed	1.2 ± 1.6	1.2 ± 2.2	0.0	0.652
Missing	9.2 ± 8.5	6.4 ± 8.7	2.8	0.072
Filled	3.7 ± 4.4	3.1 ± 3.4	0.6	0.377
Physical Functioning (PF)	48.9 ± 9.8	54.8 ± 4.9	−5.9	0.001
Role Physical (RP)	40.6 ± 9.6	45.3 ± 8.7	− 4.7	0.006
Bodily Pain (BP)	46.4 ± 11.8	52.5 ± 9.3	− 6.1	0.002
General Health (GH)	48.5 ± 10.9	54.1 ± 9.9	−5.6	0.004
Vitality (VT)	52.1 ± 10.8	56.1 ± 8.9	− 4.0	0.030
Social Functioning (SF)	47.5 ± 12.1	52.2 ± 8.1	− 4.7	0.014
Role Emotional (RE)	35.4 ± 11.2	42.2 ± 9.0	− 6.8	0.001
Mental Health (MH)	46.9 ± 12.8	50.3 ± 9.2	−3.4	0.099
Physical Component Summary (PCS)	48.3 ± 8.6	53.8 ± 6.9	−5.5	0.001
Mental Component Summary (MCS)	43.8 ± 10.8	48.0 ± 8.2	−5.2	0.019

*Independent Sample Student-t Test

**Table 3:** Mean and Standard Deviations of Oral Health Profile and Health-related Quality of Life indicators of 120 patients according to sex, Salvador, Bahia, Brazil, 2017

Indicator	Male (*N* = 64)	Female (*N* = 56)	Mean Difference	P≤*
DMFT Index- mean (SD)	10.1 ± 8.0	15.1 ± 7.8	−5.0	0.001
Decayed- mean (SD)	0.8 ± 1.4	1.5 ± 2.4	− 0.7	0.065
Missing- mean (SD)	6.3 ± 7.8	9.7 ± 9.3	−3.4	0.033
Filled- mean (SD)	3.0 ± 3.5	3.9 ± 4.4	−0.9	0.229
Physical Functioning (PF)	53.0 ± 7.6	50.4 ± 9.0	2.6	0.089
Role Physical (RP)	45.1 ± 8.2	40.3 ± 10.2	4.8	0.006
Bodily Pain (BP)	51.6 ± 10.0	46.8 ± 11.7	4.8	0.016
General Health (GH)	52.3 ± 10.3	50.0 ± 11.3	2.3	0.236
Vitality (VT)	55.8 ± 10.2	52.1 ± 9.7	3.7	0.044
Social Functioning (SF)	50.9 ± 9.3	48.6 ± 11.9	2.3	0.236
Role Emotional (RE)	39.7 ± 10.7	37.5 ± 10.8	2.2	0.247
Mental Health (MH)	52.1 ± 8.6	44.6 ± 12.7	7.5	0.001
Physical Component Summary (PCS)	52.3 ± 7.2	49.4 ± 9.2	2.9	0.057
Mental Component Summary (MCS)	47.9 ± 8.6	43.4 ± 10.6	4.5	0.013

*Independent Sample Student-t Test

**Fig. 1:**
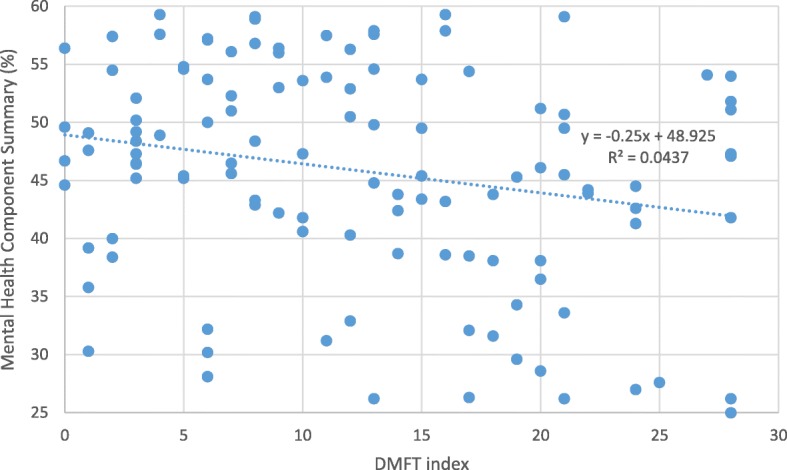
Association between Mental Health Component Summary and DMFT index in 120-HIV-infected patients

A linear regression equation estimated a significant (*p* = 0.022) decrease of 0.25 unit (%) in MCS for each unit increase in DMFT. Among depressed patients, a significant (*p* = 0.008) decrease of 0.67% in MCS for each unity increase in DMFT was estimated. (Fig. 1 and Table 4). Depressed patients showed worse oral health indicators (DFMT index; *p* ≤ 0.001 and mean Missing Teeth; *p* ≤ 0.052) and lower HRQoL domains than those without depressive symptoms. DMFT remained associated with depression (*P* < 0.005) after controlling for age, MCS, and comorbidities.

**Table 4:** Mean and Standard Deviations characteristics (Oral Health Profile and Health-related Quality of Life) of 120 patients according to depression, Salvador, Bahia, Brazil, 2017

Characteristics	Depressed (*N* = 28)	Nondepressed (*N* = 92)	Mean Difference	P≤*
DMFT Index- mean (SD)	16.9 ± 6.5	11.1 ± 8.2	5.8	0.001
Decayed- mean (SD)	1.5 ± 2.0	1.0 ± 1.9	0.5	0.217
Missing- mean (SD)	10.6 ± 8.4	7.0 ± 8.6	3.6	0.052
Filled- mean (SD)	4.8 ± 4.8	3.0 ± 3.6	1.8	0.083
Physical Functioning (PF)	48.0 ± 9.3	52.9 ± 7.7	− 4.9	0.006
Role Physical (RP)	36.6 ± 10.9	44.8 ± 8.1	−8.2	0.001
Bodily Pain (BP)	43.3 ± 11.4	51.2 ± 10.4	−7.9	0.001
General Health (GH)	47.0 ± 13.2	52.5 ± 9.7	− 5.5	0.050
Vitality (VT)	49.2 ± 9.1	55.5 ± 10.0	− 6.3	0.003
Social Functioning (SF)	44.1 ± 13.0	51.6 ± 9.2	−7.5	0.008
Role Emotional (RE)	29.2 ± 10.8	41.6 ± 9.0	−12.4	0.001
Mental Health (MH)	39.9 ± 13.6	51.2 ± 9.1	− 11.3	0.001
Physical Component Summary (PCS)	47.8 ± 9.1	51.9 ± 7.8	−4.1	0.021
Mental Component Summary (MCS)	37.1 ± 8.8	48.5 ± 8.5	−11.4	0.001

*Independent Sample Student-t Test

## Discussion

Depression is the most common prevalent neuropsychiatric symptom in HIV-1 patients [5]. According to our results, patients with depression had higher mean of missing teeth than patients without depression (*P* ≤ 0.052). A linear regression equation predicted a significant (*P* < 0.008) decrease of 0.67 unit (%) in MCS for each unit of DMFT among patients with depression. Depression can decrease the likelihood of using oral health services, and is associated with teeth loss [22].

In this study, patients with HIV had a mean DMFT of 12.4. DMFT mean in patients with HIV varies around the world, ranging from 8.7 in Australia [23], to 16.9 in Portugal [24]. In Brazil, DMFT means of 16.9, 17.64 and 18.8 have been reported [25–27]. These differences in DMFT means can be attributed to hygienic behavioral, access to dental services and socioeconomic status [28, 29]. A multivariate linear regression identified age (*P* < 0.001) and depression (*P* < 0.004) as good and independent predictors of DMFT, even after adjusting for mental health and comorbidities. Correlation between age and the mean DMFT index have also been reported not only in HIV/AIDS patients [28], but also in non-HIV/AIDS patients [30]. Older age is associated with greater frequency of dental extraction due to caries, periodontal disease, and presence of comorbidities such as diabetes, hypertension or hyperlipidemia [30].

In our study, women had systematically lower mean scores of SF-36 domains, lower MCS (*P* < 0.013) and PCS (*P* < 0.057). Our data are according to a previous study that reported females have significant lower MCS score, but not PCS [31]. Our male patients presented significantly lower mean of missing teeth than the females, differing of the results reported by in an Iranian study [28].

In a meta-analysis with 42,366 patients, from 111 studies, the prevalence of depressive symptoms ranged from 12.8 to 78.0% in HIV/AIDS patients using ART [9]. In our patients, the prevalence of depression was 23.3%, which is in accordance with the mentioned meta-analysis. The actual diagnosis of depression, as well as its previous history, have been associated with poorer HRQoL in HIV-infected patients [32]. The group with confirmed diagnosis of depression exhibited significantly lower means in all SF-36 domains and in physical and mental summary components. As expected, depression was more associated with mental health domains. The presence of major depressive disorders along patient’s life is correlated with both Physical and Mental summary scores of HRQoL [2]. Depression may also be associated with sleep disorders and appetite decrease [8].

HIV-infected patients aged 50 years or older may have multiple comorbidities and risk for cardiovascular and renal diseases [4]. In our study, at least one comorbidity was present in 51.7% of the IHV-patients. Patients without comorbidities presented lower mean DMFT. Patients with comorbidity were 4.8 years older than those without comorbidities, what may partially explain the worse mean DMFT. The group with comorbidities also presented lower means of PCS and MCS scores. The negative effect of comorbidities, specifically on the physical domains of HRQoL, has been also reported [2]. On the other hand, our patients were in use of ART for at least one year, so we can assume that they had enough time to get benefits from this medical treatment, including in their HRQoL. Improvements in physical and mental aspects of HRQoL were reported in patients using ART for one year [31].

Our study has some limitations. First, we used a cross-sectional design that have inherent methodological limitations, like the difficulty to establish the correct temporal sequence of exposure and effect. Our patients were recruited from a single reference center for HIV-infected patients. We did not take the exposure duration to ART into consideration in our analysis. The results of some clinical data were obtained from medical records. We did not have an HIV-uninfected population to compare the frequency of comorbidities. However, this is a well characterized sample that was large enough to provide insights on significant associations between oral health and HRQoL, a field with scarce data, especially in less-developed settings.

## Conclusions

In conclusion, this study found associations between poor oral health (high DMFT index) and Mental Health Component Summary in HIV-infected patients with depression. Lower health-related quality of life and poorer oral health were observed in patients with comorbidities. These findings reinforce that patients with depression should deserve especial attention to their HRQoL and oral care.
